# Biological and Genomic Characteristics of MaMV-DH01, a Novel Freshwater *Myoviridae* Cyanophage Strain

**DOI:** 10.1128/spectrum.02888-22

**Published:** 2023-01-05

**Authors:** Li-Hui Meng, Fei Ke, Qi-Ya Zhang, Zhe Zhao

**Affiliations:** a Jiangsu Province Engineering Research Center for Marine Bio-resources Sustainable Utilization, College of Oceanography, Hohai University, Nanjing, China; b State Key Laboratory of Freshwater Ecology and Biotechnology, Institute of Hydrobiology, Chinese Academy of Sciences, Wuhan, China; Connecticut Agricultural Experiment Station

**Keywords:** *M. aeruginosa* FACHB-524, MaMV-DH01, cyanophages, complete genome

## Abstract

The genomic traits of cyanophages and their potential for metabolic reprogramming of the host cell remain unknown due to the limited number of studies on cyanophage isolates. In the present study, a lytic *Microcystis* cyanophage, MaMV-DH01, was isolated and identified. MaMV-DH01 has an icosahedral head approximately 100 nm in diameter and a tail 260 nm in length. Its burst size is large, with approximately 145 phage particles/infected cell; it has a latent period of 2 days, and it shows high stability under pH and temperature stresses. Multiple infection (multiplicity of infection [MOI] 0.0001 to 100) results showed that when the MOI was 0.0001, MaMV-DH01 needed a longer time to lyse host cells. Cyanophage MaMV-DH01 has a double-stranded DNA genome of 182,372 bp, with a GC content of 45.35% and 210 predicted open reading frames (ORFs). These ORFs are related to DNA metabolism, structural proteins, lysis, host-derived metabolic genes, and DNA packaging. Phylogenetic trees based on the whole genome and two conserved genes (*TerL* and *capsid*) indicate that MaMV-DH01 is clustered with Ma-LMM01 and MaMV-DC, which are independent of other cyanophages. Collinearity analysis showed that the complete genome of MaMV-DH01 was longer than those of Ma-LMM01 and MaMV-DC, with lengths of 20,263 bp and 13,139 bp, respectively. We verified the authenticity of these excess DNA fragments and found that they are involved to various degrees in the MaMV-DH01 transcription process. Map overlays of environmental virus macrogenomic reads onto the MaMV-DH01 genome revealed that viral sequences similar to that of MaMV-DH01 are widespread in the environment.

**IMPORTANCE** A novel freshwater *Myoviridae* cyanophage strain, MaMV-DH01, was isolated; this strain infects Microcystis aeruginosa FACHB-524, and the biological and genomic characteristics of MaMV-DH01 provide new insights for understanding the mechanism by which cyanophages infect cyanobacterial blooms.

## INTRODUCTION

Cyanobacteria are abundant in freshwater and marine environments and are fundamental components of global biogeochemical cycles and primary production (1). A variety of environmental factors causing eutrophication propagate the growth of cyanobacteria on the surface and in subterranean areas of water to affect cyanobacterial blooms (2). Microcystis aeruginosa, a prominent species among cyanobacterial blooms, produces microcystins, which ultimately cause substantial economic losses in aquaculture (3–6). Recent studies have shown that cyanophages can be major determinants for controlling the population of cyanobacteria (7, 8). Therefore, biological control of *Microcystis* blooms using cyanophages has been proposed as a potential solution for the prevention of damaging cyanobacterial blooms.

Cyanophages, double-stranded DNA (dsDNA) viruses, play critical roles in regulating biomass, influencing the diversity and succession of communities, stimulating biogeochemical cycles, and mediating gene transfer among other microorganisms (9–11). Cyanophages, which regulate populations of cyanobacteria, could be used as environmentally friendly biological agents for controlling cyanobacterial blooms (12–14). Compared to marine cyanophages, which have been widely studied, there are few reports on freshwater cyanophages and, specifically, *Microcystis* phages (15). To date, only 13 *Microcystis* cyanophages have been reported, for which only 8 genomes (those of MaMV-DC, Ma-LMM01, Ma-LBP, Mic1, Me-ZS1, PhiMa05, Mae-Yong924-1, and MinS1) have been sequenced, with genome sizes under 200 kb (16–23). Only six of these reported cyanophages have been isolated from *M. aeruginosa*: Ma-LBP, Ma-LMM01, MaMV-DC, Mic1, Mae-Yong924-1, and MinS1. According to morphological characteristics, MaMV-DC and Ma-LMM01 were grouped in the *Myoviridae*, Ma-LBP and Mae-Yong924-1 were assigned to the *Podoviridae*, and Mic1 and MinS1 belong to the *Siphoviridae*.

In this study, we investigated the biological characteristics and the genomic composition of a novel freshwater cyanophage, MaMV-DH01, which was isolated from Donghu Lake in Wuhan, China. We first studied the biological characteristics of MaMV-DH01 (virion stability, growth kinetics, burst size, and host range). Based on cyanophage cultivation, the genome research of MaMV-DH01 can not only enrich the genome database of freshwater cyanophages but also aid systematic understanding of the similarities and differences between the cyanophage genomes. The application of both genomic and molecular analyses helps us to deeply understand the characteristics of MaMV-DH01. In short, this work has successfully established an infection model of culturable cyanophages in the laboratory, which lays a foundation for studying the interaction between MaMV-DH01 and M. aeruginosa FACHB-524, and accumulated data for the realization of cyanophage control of cyanobacterial bloom in the future.

## RESULTS AND DISCUSSION

### Isolation and morphology of cyanophage MaMV-DH01.

**FIG 1:**
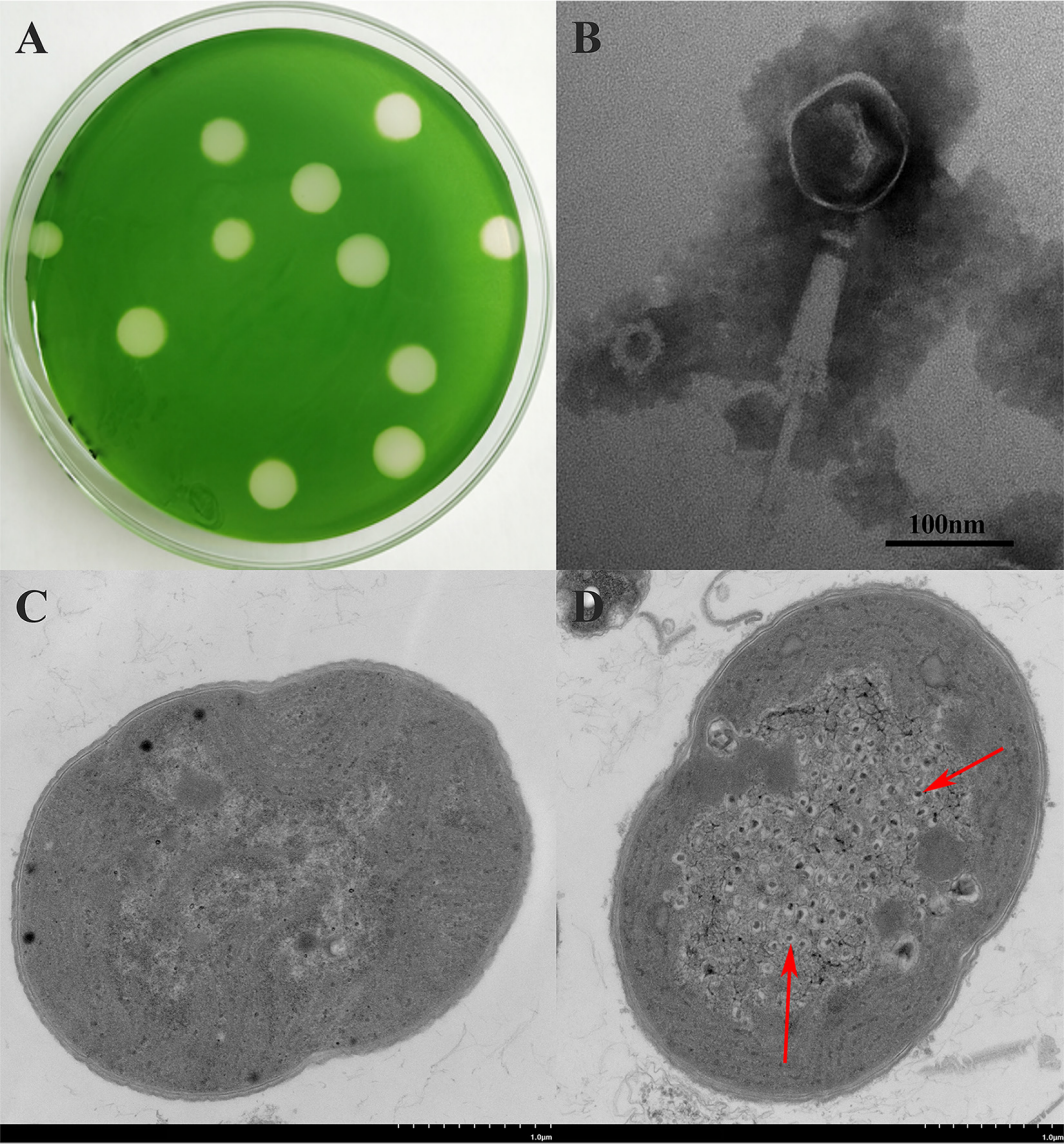
(A) Plaques of cyanophage MaMV-DH01 on the lawn of FACHB-524; (B) TEM images of cyanophage MaMV-DH01; (C) thin section of a healthy cell of FACHB-524; (D) thin section of *M. aeruginosa* FACHB-524 2 days after incubation with MaMV-DH01.

To explore the lysogenic (lytic) characteristics of MaMV-DH01, we observed plaque formation by the conventional double agar method (19). The plaque assay displayed transparent and round plaques with a diameter of approximately 5 to 6 mm on the lawns of *M. aeruginosa* FACHB-524 culture on day 7, which indicated that it was a lytic cyanophage (Fig. 1A). A transmission electron microscopy (TEM) image revealed that MaMV-DH01 had an isometric hexagonal head approximately 90 nm in diameter and a contractile tail approximately 230 nm in length (Fig. 1B). MaMV-DH01 was morphologically most similar to MaMV-DC (24); however, its head diameter was slightly greater than that of MaMV-DC (70 nm). Based on its morphology and comparisons to the current International Committee on Taxonomy of Viruses (ICTV) classification system, MaMV-DH01 belongs to the family *Myoviridae* from the order *Caudovirales*. Propagation of intracellular phage-like particles was detected in the host cells after 2 days of infection with MaMV-DH01 (Fig. 1D) compared with uninfected *Microcystis* cells (Fig. 1C), suggesting that MaMV-DH01 succeeded in entering hosts and propagating.

### Host specificity and optimal multiplicity of infection (MOI).

Generally, myoviruses show the broadest host range among the three families comprising tailed viruses, whereas podoviruses have the narrowest range (25). Interestingly, host infectivity tests showed that the myovirus cyanophage MaMV-DH01 lysed only *Microcystis* strain FACHB-524 and none of the additional 17 *Microcystis* strains or other cyanobacterial strains, which indicated that MaMV-DH01 had a narrow host range with highly constrained strain specificity (Table 1). This finding agrees with previous reports that four other *Microcystis* cyanophage strains (Ma-LMM01, MaMV-DC, PhiMa05, and Mic1) also have high host specificity (17, 20, 21, 24). Conversely, other *Microcystis* cyanophage strains showed a wide host range, including the myoviruses Mae-Yong924-1 and MinS1 and the siphovirus Me-ZS1 (3, 22, 23) (Table 2). These reports collectively reinforced the understanding of the specificity and complexity of cyanophage-host interactions and the diversity of the host range of *Microcystis* phages.

**TABLE 1:** List of cyanobacteria used for the host range test

Genus	Strain	Origin	Susceptibility
*Microcystis*	FACHB-524	Donghu Lake, China	+
	FACHB-526	Donghu Lake, China	−
	FACHB-865	Unknown, Japan	−
	FACHB-912	Taihu, China	−
	FACHB-913	Medicinal Spring Lake, China	−
	FACHB-918	Unknown, Japan	−
	FACHB-924	Unknown, Australia	−
	FACHB-927	Unknown, France	−
	FACHB-928	Unknown, Japan	−
	FACHB-936	Dianchi Lake, China	−
	FACHB-939	Tuanshan Reservoir, China	−
	FACHB-942	Unknown, China	−
	FACHB-977	Dianchi Lake, China	−
	FACHB-978	Dianchi Lake, China	−
	FACHB-1179	Donghu Lake, China	−
	FACHB-1328	Chaohu Lake, China	−
	FACHB-1338	Chaohu Lake, China	−
	FACHB-1343	Taihu, China	−
*Anabaena*	FACHB-709	Donghu Lake, China	−
	PCC7120	Unknown, France	−
	FACHB-1091	Donghu Lake, China	−
	HAB0502	Fishing pond, China	−
	HAB0984	Unknown	−
	FACHB-1198	Fishing pond, China	−
	FACHB-1199	Fishing pond, China	−
	FACHB-1246	Fishing pond, China	−
*Oscillatoria*	FACHB-528	Donghu Lake, China	−
	FACHB-1083	Donghu Lake, China	−
	FACHB-708	Donghu Lake, China	−
*Nostoc*	FACHB-713	Donghu Lake, China	−
	FACHB-973	Donghu Lake, China	−
*Planktothrix*	FACHB-1166	Fruit Lake, China	−
*Spirulina*	FACHB-971	Donghu Lake, China	−
*Phormidium*	FACHB-1136	Donghu Lake, China	−
*Chroococcus*	FACHB-193	Donghu Lake, China	−
*Synechococcus*	PCC6803	Unknown	−
*Scenedesmus*	FACHB-508	Unknown, China	−
*Eudorina*	FACHB-529	Unknown	−

**TABLE 2:** Full list of *Microcystis* cyanophages, including cyanophage MaMV-DH01

Phage name	Latent period (h)	Burst size (PFU/cell)	Classification	Host range	Length (bp)	GenBank accession no.
Ma-LBP	11.2	28	*Podoviridae*			
Ma-LMM01	6–12	50–120	*Myoviridae*	Strain specific	162,109	AB231700.1
MaMV-DC	24–48	80	*Myoviridae*	Strain specific	169,223	KF356199.1
Me-ZS1	108		*Siphoviridae*	12/15	49,665	MK069556
phiMa05	24	127	*Myoviridae*	Strain specific	273,876	MW495066.1
Mic1			*Siphoviridae*	Strain specific	96,627	MN013189.1
MinS1	36–42	34	*Siphoviridae*	19/30	49,966	MZ923504
Mae-Yong924-1			*Myoviridae*	6/14	40,325	MZ447863
MaMV-DH01	48	145	*Myoviridae*	Strain specific	182,372	OP394178

**FIG 2:**
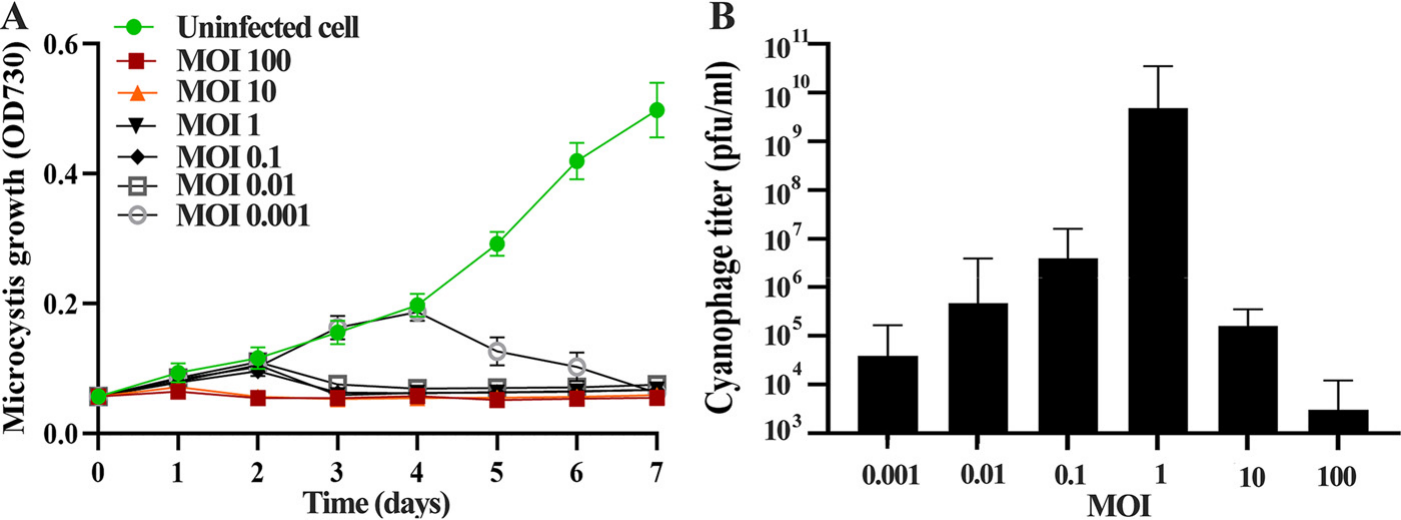
Determination of the multiplicity of infection of phage MaMV-DH01. (A) Killing time assay of cyanophage MaMV-DH01 against *M. aeruginosa* FACHB-524 at MOIs of 0.001, 0.01, 0.1, 1, 10, and 100; (B) cyanophage numbers on day 7 after *M. aeruginosa* FACHB-524 infection at MOIs of 0.001, 0.01, 0.1, 1, 10, and 100.

The MOI is the ratio of the inoculated cyanophage concentration (titer) to the host cyanobacterial concentration. It is essential for determining the optimal number of cyanophages required to kill a particular number of host cyanobacteria (26). Figure 2A presents a graph depicting the growth and lysis of cyanobacterial cells when infected with a specific lytic cyanophage under various MOIs (0.001 to 100) versus a control. Cyanophage MaMV-DH01 inhibited the growth of FACHB-524 in a concentration-dependent manner. Higher MaMV-DH01 concentrations resulted in a more rapid decrease in the number of viable FACHB-524 cells. At MOIs of 100 and 10, the bacterial host (FACHB-524) cell density decreased dramatically after 1 day of incubation (*t* test, *P < *0.05), after which the cyanobacterial population entirely collapsed within 2 days (see Fig. S1 in the supplemental material) (*t* test, *P < *0.01). Bacterial host reduction at MOIs of 1, 0.1, and 0.01 was observed after 2 days of incubation. At lower MOIs (0.001), *Microcystis* growth was significantly inhibited after 4 days of inoculation. “MOI” refers to the ratio of virus particles to host cells (27). In this study, the highest phage particle density (6.8 × 10^9^ infectious units/mL) was obtained under infection at an MOI of 1 (Fig. 2B). Cyanophages rely on the chance of contacting a cyanobacterial cell to infect and kill it. Lower MOIs may require additional time for effective phage infection, possibly resulting in the activation of host coping mechanisms for blocking phage infection (28).

### One-step growth curve and cyanophage stability.

**FIG 3:**
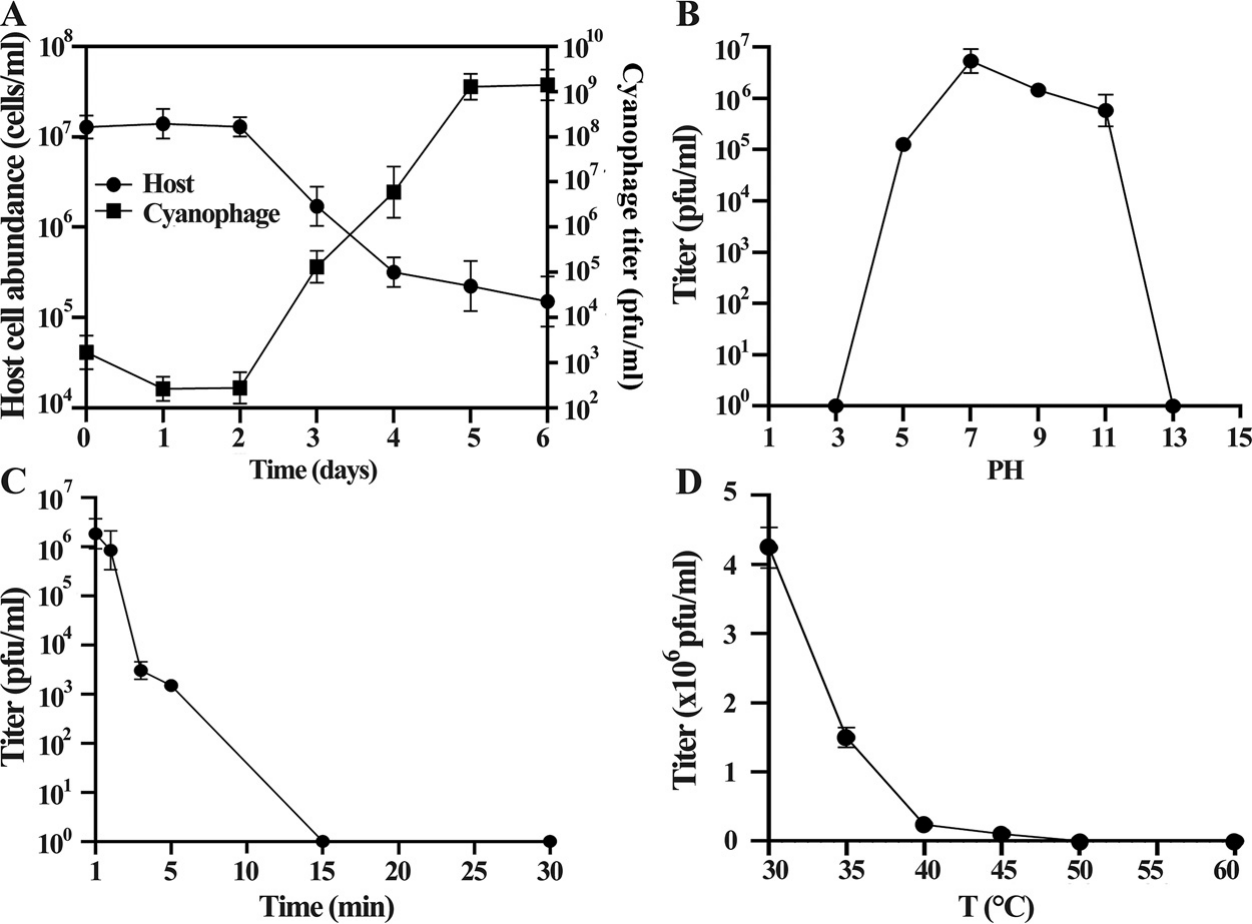
(A) One-step growth curve of cyanophage MaMV-DH01; (B) sensitivity of cyanophage MaMV-DH01 to acidic and alkaline conditions; (C) sensitivity of cyanophage MaMV-DH01 to UV light; (D) stability of cyanophage MaMV-DH01 to temperature variability. Data are the means and standard deviations of triplicate independent experiments.

To understand the growth kinetics of MaMV-DH01, a one-step growth curve experiment was performed with cyanophage at an MOI of 1 (Fig. 3A). Starting 2 days after MaMV-DH01 infection of the host, the density of host cells gradually decreased, and the cyanophage titer began to increase. This result showed that the infection latency of MaMV-DH01 may be up to 2 days. Using a 6-day infection cycle, by monitoring the decrease in viable host cells and the increase in cyanophage titer, we estimated that each infected host cell releases approximately 145 cyanophage particles at the cellular burst point. It is well known that cyanophages can infect target bacteria and enhance their infection effectiveness through self-replication in the bacterial cell (29). However, to date, only five studies have reported this type of one-step growth curve for *Microcystis* cyanophage (Table 2). Specifically, the release amounts of Ma-LBP and MinS1 were the smallest, at 28 and 34 phage particles/infected cell, respectively. It has been reported that MaMV-DC had a 2-day latent period and a burst size of approximately 80 phage particles/infected cell (16). Another *Microcystis* cyanophage, Ma-LMM01, exhibited a shorter latent period, of only 12 h, with a burst abundance of 120 viral particles even without complete host cell lysis (17). In the current study, the burst size of cyanophage MaMV-DH01 determined through the one-step growth curve was approximately 145 phage particles/infected cell, similar to that of PhiMa05 (127 phage particles/infected cell), a particularly virulent *Microcystis* cyanophage (20). These results indicate that the burst size of a cyanophage is specific to individual phage strains, which may be related to their size. Surprisingly, the burst size of S-SRP02 determined by Zhang et al. using quantitative PCR (qPCR) was estimated to be ~250 (30). This difference might arise from the different quantification methods used. Using qPCR, both infective and noninfective phages can be quantified; however, a plaque assay quantifies only infective phages. In addition, various factors, such as the size of host cells and the physiological status or growth stage of host cells, may affect the latent period and burst abundance of cyanophages (31).

The ability to survive under varied environmental conditions would critically affect phage use in biological control. Therefore, phage stability was determined under several stress conditions, including pH, UV irradiation, and temperature. MaMV-DH01 was relatively stable over the moderate pH range of 5 to 11, while no viable phage particles were detected at pH levels of <3 or >13 (Fig. 3B). UV irradiation affected the survival rate of MaMV-DH01 particles, which decreased sharply over time (Fig. 3C); for example, following 1 min of UV irradiation, the survival rate was only 35%, and after UV irradiation for 15 min, the cyanophage was essentially inactivated, indicating sensitivity to this type of radiation. Considering thermal stability, after treatment at 30°C, 35°C, 40°C, 45°C, 50°C, and 60°C for 1 h, the titer of cyanophage decreased continuously with increasing temperature starting at 35°C, with the phage titer being completely lost at 50°C (Fig. 3D). These results showed that MaMV-DH01 was sensitive to highly acidic and basic conditions (pH <3 or >13) and to high temperatures (>50°C), consistent with previous research results on the effects of environmental stressors on PhiMa05 (20). Primary environmental parameters, such as water temperature and pH, have a significant effect on cyanobacterial growth, abundance, and geographic distribution, which correspondingly affect cyanophage interaction, stability, and infectivity (31). The optimal growth temperature for most cyanobacteria is 25 to 30°C, and the optimal growth pH level is at neutral or weakly alkaline levels, so cyanophages and their host cyanobacteria may have similar optimal growth conditions (32). Examining the effects of these abiotic factors on the growth of cyanophages will allow us to better understand cyanobacterial algal blooms and, therefore, how to use bacteriolytic cyanophages for biological control.

### Genome features and analysis.

**FIG 4:**
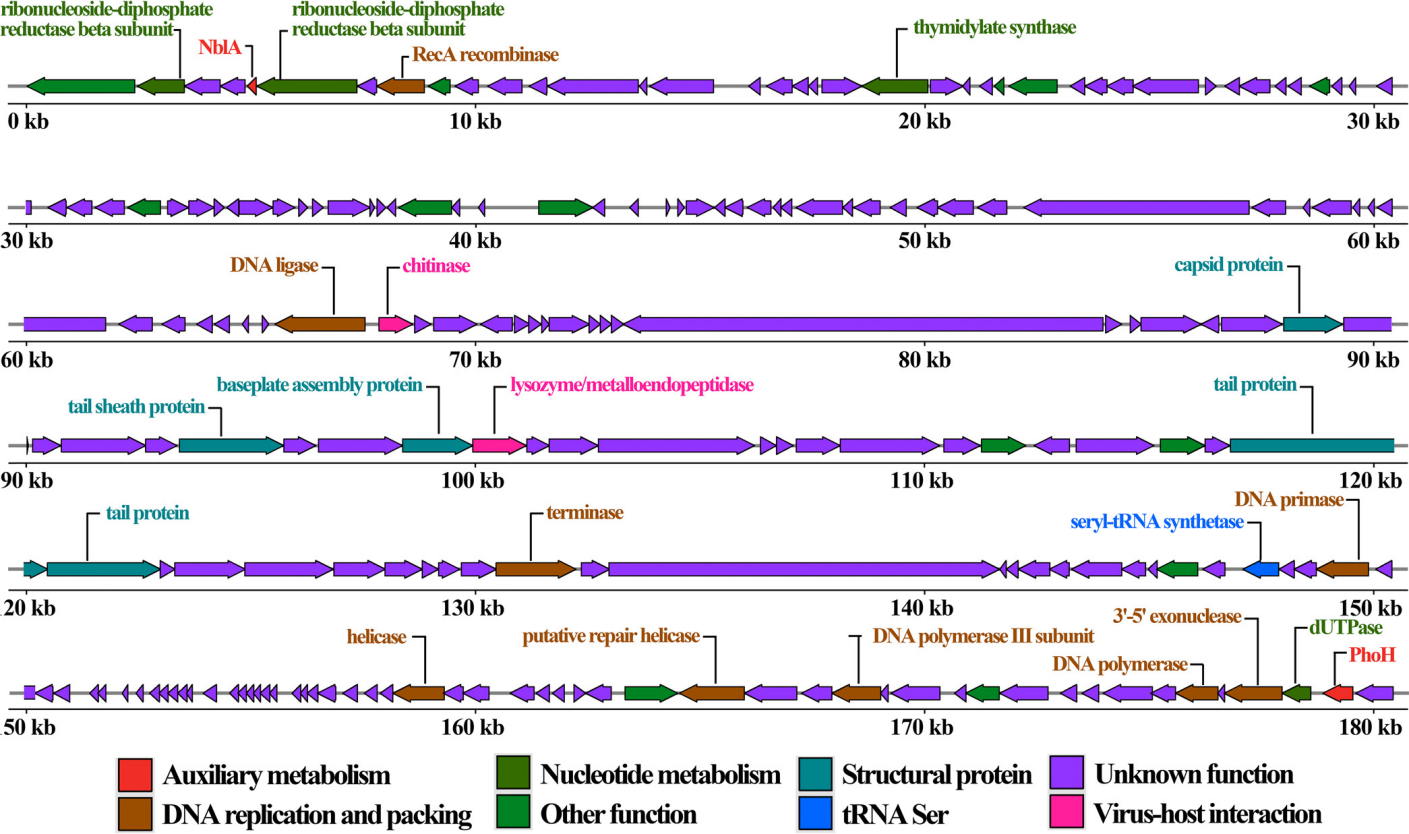
The genome map of cyanophage MaMV-DH01. Arrows indicate the size, position, and orientation of 210 ORFs. Genes within different functional categories are indicated by the colors indicated at the bottom.

MaMV-DH01 consists of a linear double-stranded DNA genome 182,372 bp in length and with a GC content of 45.35% (Fig. 4). A total of 210 ORFs were predicted in the MaMV-DH01 genome, with 69 genes on the plus strand and 141 genes on the minus strand (Table S5). In addition, the genome of MaMV-DH01 lacks RNA synthesis-related enzymes such as RNA polymerase (Table S5). These results showed that the proliferation of MaMV-DH01 in the host relies on host transcription and translation metabolic pathways.

Homology alignments to the NR database were applied to functionally annotate the 210 ORFs. Only 37 ORFs showed homology to genes with a known function (Table S5); of the total, 157 were assigned to hypothetical proteins, and 16 had no homology to sequences within the database. This high rate (82%) of unknown function may be the result of our currently poor level of knowledge about the genomes of cyanophages. Bioinformatics analyses revealed that the functionally annotated ORFs were separated into 5 groups comprising structural protein, DNA replication, and packaging genes; nucleotide metabolism genes; lysis-related genes; and auxiliary metabolism-related genes (AMGs) (Fig. 4).

**(i) Structural proteins.** In addition to the major capsid protein (ORF108) (Table S5), other sequences encoding known phage structural proteins were found in the MaMV-DH01 genome, including tail collagen protein (ORF20), putative phase tail sheath protein (ORF113), putative base plate assembly protein (ORF116), tail protein (ORF131 and ORF132), and a protein that determines the length of the tail. The majority of MaMV-DH01 structural proteins are similar to those found in MaMV-DC (16). Obtaining a more thorough understanding of structural proteins in MaMV-DH01 will require mass spectrometry analyses.

**(ii) DNA replication- and packaging-related genes.** Eight MaMV-DH01 genes involved in DNA replication, recombination, repair, and packaging were predicted in this study. ORF183 and ORF192 were predicted to encode helicase and putative A repair helicase, respectively. They are necessary to ensure proper regulation of cyanophage DNA replication initialization (33). MaMV-DH01 also encodes DNA polymerase (ORF205) and DNA polymerase III subunits (ORF195), of which the latter can replicate the host-related protein gene and express its required protein during phage replication. ORF207 was annotated as a 3′-to-5′ exonuclease, which belongs to a broad family of 5′ kinase/3′ phosphatases, enzymes with nucleic acid modification capabilities (34). In addition, MaMV-DH01 also encodes a variety of enzymes, such as RecA recombinase (ORF8), DNA primase (ORF156), and DNA terminal enzyme (ORF141), and participates in replication, repair, and recombination. An important role of terminal enzymes is to specifically cut viral DNA ligands to form a unit-length genome and package it into the capsid (35).

**(iii) Lysis-related genes.** In general, lysozyme and holin homologs are commonly found in cyanophages and are believed to be functional genes for stimulating cell lysis (17, 36, 37). We detected two genes in MaMV-DH01 (ORF91 and ORF117), which are involved in host cell lysis. The putative Zn-dependent peptidase (ORF117) was reported to cleave N-acetylmuramic acid of peptidoglycan, resulting in cell wall lysis (38). Interestingly, no homologs of holin could be found, but chitinase (ORF91) was annotated within the MaMV-DH01 genome. Chitinases of *Chlorella* virus (belonging to the hydrolase family) have been reported to play an important role in the lysis of algal cells (39, 40). Meanwhile, chitinase also exists in the genomes of cyanophages MaMV-DC and Ma-LMM01, suggesting that it may be a key gene responsible for cell lysis (17, 24). This suggests that the muscle tail cyanophage represented by MaMV-DH01 and MaMV-DC, Ma-LMM01, may adopt the same cleavage strategy, which is different from others previously discovered.

**(iv) Auxiliary metabolism-related genes.** During the coevolution of viruses and hosts, specific genes beneficial to viral infection and replication have likely been co-opted from the host genome, resulting in what has been called auxiliary metabolic genes (41). For example, the proteins encoded by AMGs are crucial for energy metabolism (photosynthesis, phycobilisome degradation, carbon metabolism, phosphorus utilization, and nucleotide biosynthesis), providing ATP (or reducing power) for nucleotide biosynthesis and phage genome replication (42–45). Generally, marine cyanophages encode more AMGs, such as S-SZBM1, which can encode up to 25 auxiliary metabolic genes (46), while freshwater cyanophages generally have fewer AMGs. In this study, we did not find AMGs related to photosynthesis in the MaMV-DH01 genome. However, the two AMGs predicted in the MaMV-DH01 genome were ORF5, encoding phycobilisome degradation protein NblA, and ORF209, encoding phosphate starvation-inducible protein. Among them, the amino acid sequence of MaMV-DH01 NblA was highly homologous with the host phycobilisome cleavage protein, suggesting that the cyanophage may have obtained it through horizontal gene transfer from the host.

**(v) Nucleotide metabolism-related genes.** In addition to auxiliary metabolic genes, cyanophages also encode enzymes related to nucleotide metabolism to assist in the process of viral genome replication. In the MaMV-DH01 genome, ORF2 and ORF6 were found to be homologous to ribonucleotide-diphosphate reductase (RNR) subunits beta and alpha, respectively. The RNR gene product can reduce ribonucleotide diphosphate to deoxyribonucleotide diphosphate, a precursor of DNA (47). RNRs generally exist in the reported genomes of *Myoviridae* cyanophages and are considered essential for the rapid replication exhibited by lytic cyanophages (47). Thus, cyanophages can use RNRs to degrade host DNA and provide building blocks for synthesizing the genome of phage progeny. In addition, the MaMV-DH01 genome contains genes related to nucleotide metabolism, including thymidylate synthase (ThyX ORF21) and uracil deoxyribonucleoside triphosphatase (dUTPase ORF208). The importance of ThyX in phage genome replication has been demonstrated in double-stranded DNA viruses (48). However, determining whether dUTPase is involved in the replication of the cyanophage genome requires further research.

### Phylogenetic analysis and genome comparison.

**FIG 5:**
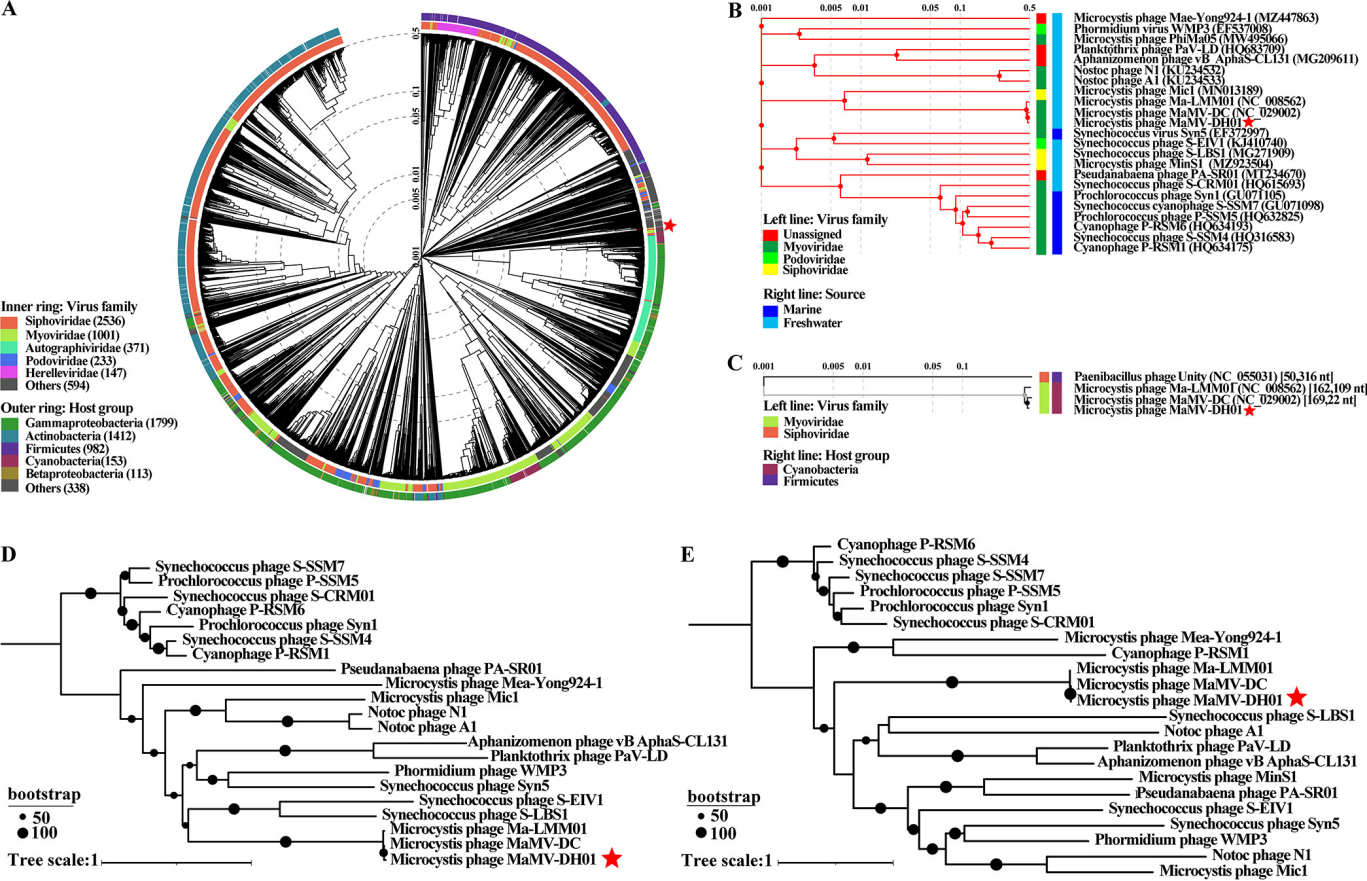
Phylogenetic analysis. (A, B, and C) Phylogenetic analysis with other related phages using the genome-wide sequence similarities computed by tBLASTx. The whole proteomic tree was generated by the ViPTree server. (D) ML phylogenetic tree with other related phages based on the amino acid sequences of major capsid proteins. (E) ML phylogenetic tree with other related phages based on the amino acid sequences of the terminase large subunit. The trees were constructed by iq-tree with the ML method and 1,000 bootstrap replicates. The percentages of replicate trees in which the associated taxa clustered together in the bootstrap test are shown next to the branches. The red star represents the phage found in this study.

To obtain a global view of the evolutionary position of MaMV-DH01 among the phages, we constructed a proteomic tree (Fig. 5A) using the reference genomes of 4,941 phages with Viptree as previously described (49). Then, onto the proteomic phylogenetic tree, we overlaid the complete genome sequences of 22 cyanophages, including 6 *Microcystis* cyanophages and 18 representatives of the 4 cyanophage families as defined in the most recent classification by the ICTV (Fig. 5B). The final proteomic tree showed that MaMV-DC and Ma-LMM01 have the closest genetic relationship with MaMV-DH01 and cluster onto the branch of cyanomyoviruses (Fig. 5B and C). The phylogenetic analyses of MaMV-DH01 and other selected dsDNA viruses performed based on the capsid protein and terminase large subunit sequences using the maximum likelihood (ML) method showed similar branching positions of MaMV-DH01 (Fig. 5D and E). In both the whole-genome evolutionary tree and the conserved gene evolutionary tree, MaMV-DH01, MaMV-DC, and Ma-LMM01 clustered onto a single branch, which was completely independent of other freshwater and marine cyanophages, indicating that the three freshwater muscle tail (contractile) phages isolated from different regions may represent a new and distinct group of *Myoviridae* cyanophages.

**FIG 6:**
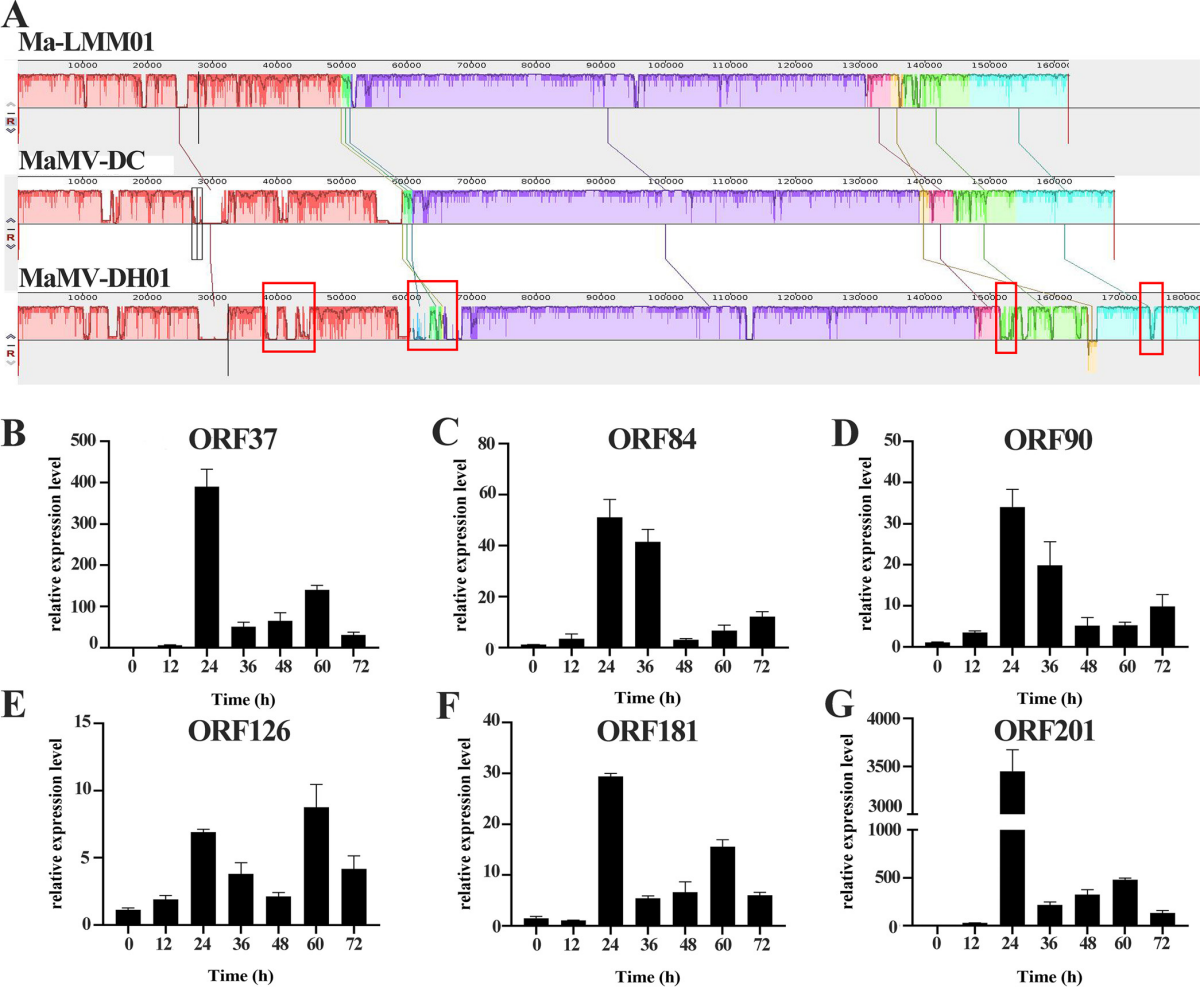
(A) Genomic collinearity of MaMV-DH01 with MaMV-DC and Ma-LMM01. The colored collinear blocks indicate homologous regions between genome sequences, while the height of the similarity profile in the colinear blocks indicates the average level of conservation in the regions of the genome sequence. The red square represents the insertion region in the MaMV-DH01 genome. (B) Relative abundance of MaMV-DH01 ORF37 mRNAs. (C) Relative abundance of MaMV-DH01 ORF84 mRNAs. (D) Relative abundance of MaMV-DH01 ORF90 mRNAs. (E) Relative abundance of MaMV-DH01 ORF126 mRNAs. (F) Relative abundance of MaMV-DH01 ORF181 mRNAs. (G) Relative abundance of MaMV-DH01 ORF201 mRNAs.

MaMV-DC and Ma-LMM01 share the highest similarity to MaMV-DH01, with nucleotide identities of 97.3% and 96.4%, respectively (Table S6). Therefore, the genomes of MaMV-DC and Ma-LMM01 were compared with that of MaMV-DH01. The genome alignment of MaMV-DH01 with *Microcystis* cyanophages (Ma-LMM01 and MaMV-DC) is shown in Figure 6A. Three colinear blocks are shared among MaMV-DH01, Ma-LMM01, and MaMV-DC with different arrangements and lengths. The three genomes were found to be highly similar in specific proteins encoded by the conserved genes, including DNA polymerase, terminase large subunit, and the major capsid protein, with 75.31 to 93.65% identity at the amino acid level. The principal genomic difference among MaMV-DH01, MaMV-DC, and Ma-LMM01 is the insertion of host or other microbial DNA, the size of which represents approximately 7 % of their total genomes (e.g., mainly ORF13-15, ORF30-39, and ORF67-68). These inserted or absent gene fragments may point to specific variations between MaMV-DH01, MaMV-DC, and Ma-LMM01 regarding host infectivity.

Next, we verified the gene sequence of the insertion region in the MaMV-DH01 genome. The results showed that the verified ORF did exist in the MaMV-DH01 genome (Fig. S2), and the sequencing results were consistent with the genome sequencing results. In addition, we also randomly evaluated whether six genes were involved in the transcription process of MaMV-DH01 using qPCR. The results revealed various degrees of transcription during a 72-h infection period (Fig. 6B to G). These results suggest that the genes in the insertion region are involved in the MaMV-DH01 infection process, and we speculate that they may be functional genes. However, more research is needed to further verify specific gene functions.

### MaMV-DH01-like sequences in the environment.

**FIG 7:**
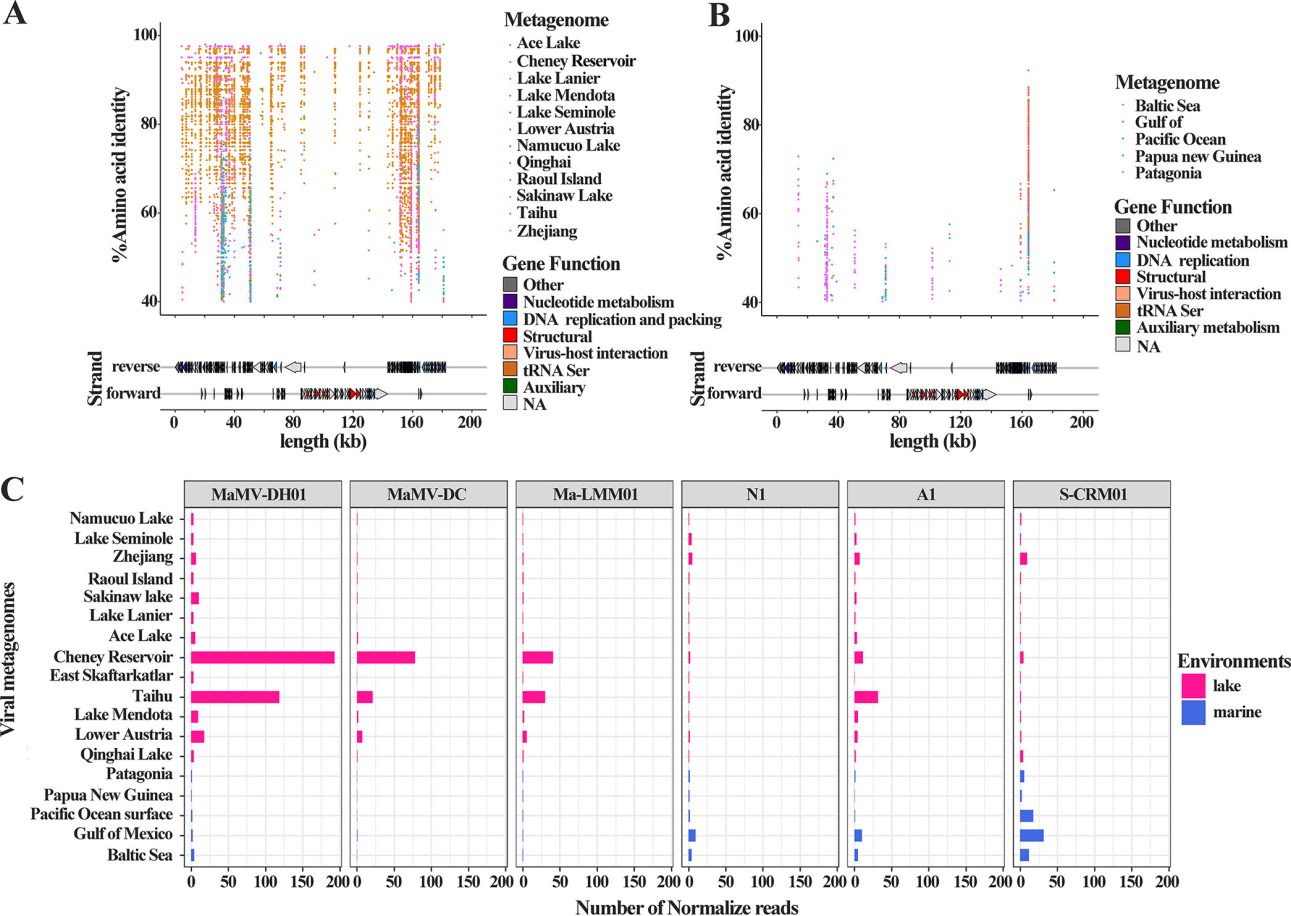
Prevalence of MaMV-DH01-like sequences in environmental viral metagenomic data. (A and B) Fragment recruitment of reads from environmental viral metagenomic data (Table S3) onto the genome of MaMV-DH01. Each horizontal line represents a read recruited from one of the following publicly available metagenomics data sets: freshwater viral metagenomic data sets (A) and marine viral metagenomic data sets (B). (C) Normalized total number of recruited reads for MaMV-DH01 (amino acid identity >40%) and other phages across environments. The total number of hits to phage was normalized by dividing by the length of the phage genome (in kilobases) and the size of the database (number of reads recruited per kb of genome/size of the database in gigabytes), which provides a normalized measure to compare recruitment by distinct size contigs versus several metagenomic data sets.

To explore the possibility of the existence of MaMV-DH01-like viruses in the aquatic environment, the genome reads of MaMV-DH01 were recruited from the metagenomic data sets of marine and freshwater environments (Fig. 7A and B). The MaMV-DH01-like sequences have been detected in almost all freshwater metagenomic data sets, although the relative abundance of each ORF varies among environments, with higher abundance in Lake Mendota, Taihu, and Cheney Reservoir (Fig. 7A). In general, a total of 203 ORFs were mapped to the freshwater metagenomics, of which 91 ORFs were detected in multiple freshwater metagenomes, and 13 ORFs had higher abundance in those environments (Table S7). However, few reads were mapped to those ORFs of MaMV-DH01 from the marine metagenomics (Table S8). This result showed that MaMV-DH01-like sequences were more common in freshwater than in marine water. Interestingly, most reads from Cheney Reservoir were recruited to the MaMV-DH01 genome (identity from 40% to 100%), and those ORFs were enriched into DNA replication, repair (ORF90 and ORF195), and cleavage (ORF91 and ORF117) (Fig. 7A), which indicated the possible presence of a novel freshwater cyanophage closed to MaMV-DH01 in Cheney Reservoir water. Those results mean that aquatic viruses with a metabolism and release mechanism similar to those of MaMV-DH01 are widespread in the aquatic environment.

Similar to selected freshwater *Myoviridae* cyanophages (e.g., MaMV-DC, Ma-LMM01, N1, S-CRM01, and A1), MaMV-DH01-like viruses seem to be widespread in freshwater (Fig. 7C). In addition to freshwater lakes, MaMV-DH01 seems to also be enriched in marine metagenomes of the Baltic Sea, Gulf of Mexico, Pacific Ocean surface waters, Papua New Guinea, and Patagonia. However, compared with that in freshwater environments, the abundance of the six freshwater cyanophages in marine environments was low. This is consistent with the freshwater natural history of these freshwater cyanophages (MaMV-DC, Ma-LMM01, N1, S-CRM01, and A1).

### Conclusion.

The present study describes the characteristics and genome of MaMV-DH01, which was the second cyanophage reported to specifically infect *M. aeruginosa* FACHB-524. MaMV-DH01 is a *Myoviridae* cyanophage possessing several genes, including AMGs, structural protein-encoding genes, and other genes involved in phage DNA replication and host cell lysis. MaMV-DH01 has a large burst abundance, rapid killing capability, and high stability under pH and temperature stresses. These results provide new insights into possibilities for the biological control of cyanobacteria using cyanophages. Furthermore, evidence from metagenomic mapping suggests that the sequence of MaMV-DH01-like exists widely in aquatic environments and is likely to be a key component in controlling and structuring *M. aeruginosa*-related populations in marine and freshwater environments. Although viral infections have been considered a promising strategy for controlling cyanobacterial populations, further research is needed to explore the safety of their potential, specifically whether MaMV-DH01 application may be appropriate.

## MATERIALS AND METHODS

### Isolation and morphological observation of MaMV-DH01.

Surface water samples were collected from Donghu Lake in Wuhan City. The samples were centrifuged at 10,000 × *g* for 20 min at 4°C and filtered sequentially through 0.45-μm and 0.22-μm pore filters (Millipore, Bedford, MA, USA). Aliquots (3 mL) of the filtrates were mixed with *M. aeruginosa* FACHB-524 cultures at the exponential phase (volume ratio of 1:2) and cultured in an incubator under a light-dark cycle of 12:12 h with a constant day illumination of 40 μmol photons m^−2^ s^−1^ at 25°C for 10 to 20 days until the cultures were etiolated (50). The yellowing lysate was centrifuged, filtered, and applied to the next round of enrichment, as described above. The enrichment was performed for three rounds. A pure cyanophage strain was obtained by five serial single-plaque isolations using the double-layer agar method (3). A single plaque was removed from the plate, mixed with 3 mL of exponentially growing FACHB-524, and cultured in an illuminated incubator for 7 to 10 days. The yellowed and clarified culture suspension was centrifuged (6,000 × *g*, 20 min) and filtered (0.45 μm and 0.22 μm). Amplification culture was performed via cocultivation of *M. aeruginosa* FACHB-524 and the filtrate containing the *Microcystis* cyanophage MaMV-DH01 (at a volume ratio of 5:1) until the culture turned yellow (at approximately 7 days).

Cyanophage size and morphology were determined by negatively stained images. A suspension of MaMV-DH01 was dripped onto 400-mesh copper grids, stained with 2% uranium acetate (Sigma-Aldrich, St. Louis, MO, USA), and observed at 80 kV using a JEOL JEM-2010 transmission electron microscope. In addition, as previously described, ultrathin sections were prepared for electron microscopy (16). Briefly, cyanobacterial cells infected with cyanophages for 2 days were collected and fixed with glutaraldehyde, after which they were dehydrated, embedded, and sectioned. Ultrathin sections were observed with a JEOL JEM-2010 transmission electron microscope.

### Host range and multiplicity of infection (MOI) test.

We obtained 38 cyanobacterial strains (Table 1) from the Freshwater Algae Culture Bank, Institute of Hydrobiology, Chinese Academy of Sciences, Wuhan, China. Two-hundred-microliter aliquots of the MaMV-DH01 suspension were added to exponential-phase cultures (1 mL) of the 38 cyanobacterial strains in triplicate in 24-well cell culture plates and incubated in an illuminated incubator. The phage suspension was replaced with BG11 medium in the negative-control groups. Infectivity was determined by a reduction in the reading for optical density at 730 nm (OD_730_) compared to the control (51). The cultures without lysis after 14 days were considered nonsusceptible hosts (16).

In this analysis, *Microcystis* killing by cyanophage was carried out in 50-mL conical flasks. Different amounts of cyanophage solutions were added to flasks containing 15 mL of the FACHB-524 culture (10^6^ CFU/mL) to achieve MOIs of 0.001, 0.01, 0.1, 1, 10, and 100. The cultures were incubated for 7 days under the previously described conditions. Cyanobacterial density was measured daily using optical densitometry (LUMIstar Omega, Germany) at 730 nm (52). After 7 days of incubation, the cultures were centrifuged at 10,000 × *g* for 15 min, and the supernatant was used to determine the titer of MaMV-DH01 under different MOI conditions by the most likely number (MPN) (infectious units per milliliter) (53). The highest phage titer provided by specific MOIs indicates the optimal ratio between phage particles and host cells, yielding the optimal MOI. The experiment was repeated in triplicate.

### One-step growth curve and phage stability under environmental stress.

A one-step growth experiment was conducted to evaluate the latent period, burst time, and burst abundance of the cyanophage (16). Ten milliliters of the cyanophage (10^8^ PFU/mL) was added to 50 mL of the log-phase host culture (10^7^ cell/mL), and the mixture was incubated for 7 days under the conditions described above. Samples were collected at 1, 2, 3, 4, 5, 6, and 7 days. All of the 1-mL samples were centrifuged (6,000 × *g*, 10 min) and filtered (using 0.45-μm and 0.22-μm filters), and titration was performed using the MPN method. The burst abundance was calculated as the number of liberated phage particles minus the number of unadsorbed cyanophage particles divided by the number of initial bacteria (54). In addition, the density of the cyanobacterial cells was determined daily using a hemocytometer (Shanghai Medical Optical Instrument Plant, Shanghai, China) (16).

Temperature, pH, and UV sensitivity assessments were performed. (i) Different pH values in aliquots of cyanophage stock solution (7.3 × 10^6^ PFU/mL) adjusted by 5 M HCl and 5 M NaOH were created (pH values of 3, 5, 7, 9, 11, and 13), and the solutions were incubated for 12 h at 25°C. (ii) The aliquots of cyanophage stock solutions were incubated at 30°C, 35°C, 40°C, 45°C, 50°C, and 60°C for 1 h and then placed on ice for 5 min. (iii) Aliquots of cyanophage stock solutions were irradiated under a UV lamp (253.7 nm), and samples were collected at different time points (0, 1, 3, 5, 15, and 30 min). To detect the cyanophage viability after treatment with different abiotic factors, the host infection was performed under normal conditions (40 μmol photons m^−2^ s^−1^ at 25°C), and the titer of bacteriophage was tested using the MPN assay. Untreated cyanophage solution was used as a control, and each of the treatments was repeated three times. One-way analysis of variance (ANOVA) and Duncan’s new multiple-range tests were performed in SPSS 13.0 to determine statistical significance. GraphPad Prism (8.0.2) was used for curve plotting and additional statistical analysis of the data.

### DNA extraction, purification, and sequencing.

A total of 400 mL of lysates was centrifuged at 6,000 × *g* for 20 min and filtered through a 0.22-μm nitrocellulose filter. To remove free nucleic acids, the lysate was treated with DNase I (B618252; Sangon Biotech, Shanghai, China). The filtered lysate was centrifuged at 5,000 × *g* with a 100-kDa-molecular-weight (MW) cutoff in ultrafiltration centrifugal tubes (Amicon Ultra15 centrifugal filter units; Millipore) at 5,000 × *g* to a final volume of 1 mL. Cyanophage genome extraction was performed with the TIANamp virus DNA/RNA kit (Tiangen, China) according to the manufacturer’s instructions. DNA quality was checked using electrophoresis (sharp single DNA band) and a NanoDrop spectrophotometer (MaestroGen Inc.). Insertion libraries (400-bp paired-end [PE250]) were constructed with Illumina TruSeq sample V preparation reagents according to the manufacturer’s instructions and sequenced using an Illumina NovaSeq 6000 instrument (150 bp × 2; Shanghai Biozeron Co., Ltd.).

### Genome annotation and phylogenetic analysis.

The open reading frames (ORFs) were predicted with GeneMarkS and annotated using BLASTp against the nonredundant (NR in NCBI), Swiss-Prot (http://uniprot.org), KEGG (http://www.genome.jp/kegg/), and COG (http://www.ncbi.nlm.nih.gov/COG) databases. tRNA was identified using tRNAscan-SE (v2.0).

A phylogenetic tree based on whole-genome sequences was constructed using the ViPTree (https://www.genome.jp/viptree/; 20 April 2022) (49) and VIRIDIC tools (http://viridic.icbm.de/; 20 April 2022). The representative viruses were selected to build a phylogenetic tree. Meanwhile, the large terminase subunit (*TerL*) and major capsid protein were compared phylogenetically with those from other cyanophages (Table S1) using MAFFT L-INS-i (v7.294b) with default parameters (55). Then, the aligned protein sequences were trimmed using trimAl (v1.4) with the parameter “-automated1” to remove the poorly aligned regions (56). Subsequently, a maximum likelihood phylogenetic tree was constructed using iq-tree (v1.6.12) with “MFP” to select the optimal model with 1,000 replicates (“-m MFP -bb 1000 -alrt 1000”) (57).

### Genome comparative analysis and gene sequence verification.

The average nucleotide identity (ANI) was calculated using OrthoANI (Average Nucleotide Identity by Orthology) (58) and JSpeciesWS Online Service (59). Multiple genome alignment for the analysis of genomic synteny was performed using the progressive Mauve (60) plugin in Geneious software (version 2020.1.2). Genomic structural variation between MaMV-DH01 and MaMV-DC or Ma-LMM01 was analyzed using mummer software. A full list of cyanophage genomes can be found in Table S2.

Based on MaMV-DH01 genome sequencing data, 15 pairs of primers were designed by Primer Premier 5.0 (Table S3) to confirm the authenticity and accuracy of the sequence. Each PCR system contained 2 μL of genomic DNA (50 ng/μL), 1 μL each of forward primer and reverse primers (10 mM), 25 μL of high-fidelity DNA polymerase mixture (TaKaRa, Dalian, China), and 22 μL of double-distilled water (ddH_2_O) in a total reaction volume of 50 μL. The PCR conditions were as follows: initial denaturation at 95°C for 10 min; 35 cycles of 95°C for 30 s, 60°C for 30 s, and 72°C for 2 min; and a final extension at 72°C for 10 min. All the PCR products were electrophoresed on a 1% agarose gel, and the target fragments were recovered with a DNA extraction kit (Solarbio, Beijing, China) for sequencing.

### Quantitative real-time PCR.

Cells (1 to 1.5 mL) were collected at 0, 12, 24, 36, 48, 60, and 72 h postinfection and were centrifuged at 15,000 × *g* and 4°C for 10 min. Cells were flash-frozen in liquid nitrogen and stored at −80°C until RNA extraction. Total RNA was isolated from the tissues using TRIzol reagent (Invitrogen, Carlsbad, CA, USA) according to the manufacturer’s protocol and then treated with RNase-free DNase I (TaKaRa, Dalian, China) to eliminate genomic DNA contamination. Approximately 1 μg of RNA was used for first-strand cDNA synthesis with a reverse transcriptase Moloney murine leukemia virus (MMLV) kit (TaKaRa, Dalian, China) according to the manufacturer’s instructions. Quantitative real-time PCR (qPCR) was conducted using ChamQ Universal SYBR qPCR SuperMix (Vazyme, Nanjing, China) following the manufacturer’s instructions to detect the expression level of the selected gene at different time points of infection. Primers were designed using Primer Premier 5.0 (Table S3). The host *rbcl* gene was selected as the reference gene. Amplification was performed via an initial denaturation step at 94°C for 60 s, 40 cycles at 94°C for 15 s, 60°C for 15 s, and 72°C for 45 s, and a final extension at 72°C for 10 min. Relative gene expression levels were calculated by the threshold cycle (2^−ΔΔ^*^CT^*) method in triplicate samples.

### Recruitment of reads to metagenomics.

The presence of viral sequences similar to the MaMV-DH01 sequence in aquatic environments was investigated by recruiting viral metagenomics data onto the genome of MaMV-DH01. In total, 247.8 gigabytes of freshwater metagenome data and 157.7 gigabytes of marine metagenome data were used (Table S4). Briefly, metagenomic data were first made into a BLAST nucleotide database and queried with the predicted protein sequence of MaMV-DH01 using tBLASTn (E value of ≤ 10^−5^; max_target_seqs = 1; amino acid identity > 40%), which performed six-frame translation of the subject nucleotide sequence into a protein sequence. Metagenomics nucleotide reads with a BLAST hit to MaMV-DH01 were then extracted from each metagenome and used as a query to BLAST search (BLASTx; E value of ≤ 10^−5^; max_target_seqs = 1) against a viral protein database containing predicted proteins of MaMV-DH01 phage and another 4,523 bacteriophage genomes from the NCBI Reference Sequence Database (RefSeq; released on 8 January 2022). If the best hit was related to MaMV-DH01 instead of the other phages, it was recruited as a viral sequence similar to MaMV-DH01 and mapped onto the genome of MaMV-DH01. Finally, the recruited reads were mapped to the MaMV-DH01 genome based on their percent amino acid identity using ggplot2 in R. The total number of hits to MaMV-DH01 was normalized by dividing by the length of the MaMV-DH01 genome (in kilobases) and the size of the metagenome (number of reads recruited per kilobase of genome/size of the database in gigabytes), which provides a normalized measure to compare recruitment by differently sized contigs versus several metagenomes. Similar recruitment analysis was also conducted for other phages (MaMV-DC, Ma-LMM01, N1, S-CRM01, and A1).

### Data availability.

The raw data and whole-genome sequence are available from the NCBI under accession numbers PRJNA775671 (BioProject) and OP394178 (GenBank), respectively.
