# Supplementary material for: Biological and Genomic Characteristics of MaMV-DH01, a Novel Freshwater Myoviridae Cyanophage Strain
Source: Microbiol Spectr. 2023 Jan 5;11(1):e02888-22. doi: 10.1128/spectrum.02888-22 (PMC9927357; doi:10.1128/spectrum.02888-22)
Supplement: Supplemental file 1 — Fig. S1 and S2. Download spectrum.02888-22-s0001.pdf, PDF file, 0.2 MB [file spectrum.02888-22-s0001.pdf]

Supplementary Material

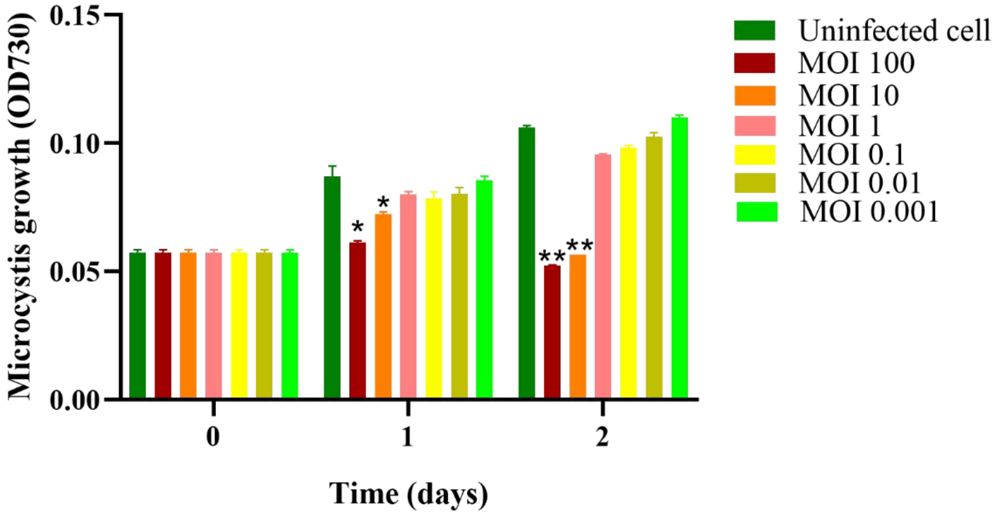

Figure S1**:** Killing time assay of cyanophage MaMV-DH01 against M. aeruginosa FACHB-524 at MOIs of 0.001, 0.01, 0.1, 1, 10, and 100 on days 1 and 2. The "*" (t test, p < 0. 05) and "**" indicates statistical significance (t test, p < 0.

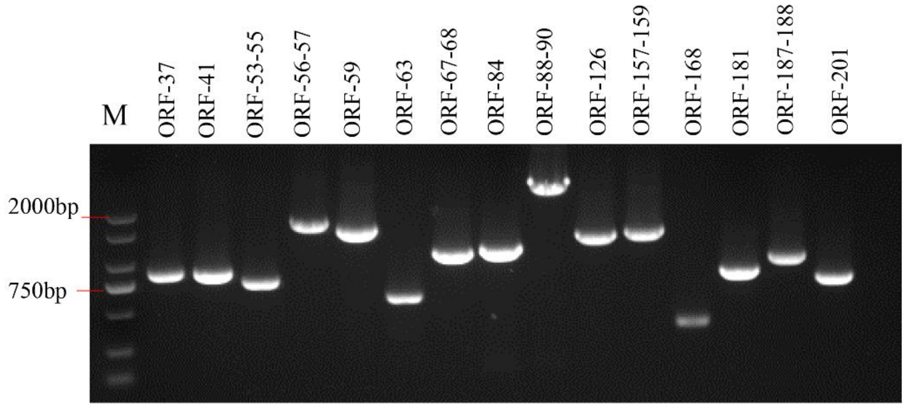

01).

Figure S2: Nucleic acid products of putative ORFs separated on 1% agarose gels. M: 2-kbp marker.
